# Diaqua­[5,5′-dicarb­oxy-2,2′-(propane-1,3-di­yl)bis­(1*H*-imidazole-4-carboxyl­ato)]nickel(II) dihydrate

**DOI:** 10.1107/S1600536811024391

**Published:** 2011-06-25

**Authors:** Guang-Hua Jin, Xifeng Li, Chunyue Hu, Lixin Hu

**Affiliations:** a Department of Chemistry, Zhengzhou University, Zhengzhou 450052, People’s Republic of China; b Pharmacy College, Henan University of Traditional Chinese Medicine, Zhengzhou 450008, People’s Republic of China

## Abstract

In the title complex, [Ni(C_13_H_10_N_4_O_8_)(H_2_O)_2_]·2H_2_O, the Ni^2+^ cation is six-coordinated by two N atoms and two O atoms from the tetra­dentate anion in equatorial positions and by two water O atoms in axial positions, leading to a distorted octa­hedral environment. The central C atom of the propanediyl unit is disordered over two sites in a 0.531 (6):0.469 (6) ratio. In the crystal, adjacent mol­ecules are linked through O—H⋯O and N—H⋯O hydrogen-bonding inter­actions into a three-dimensional network.

## Related literature

For background to complexes based on 1*H*-imidazole-4,5-dicarb­oxy­lic acid, see: Baures *et al.* (2002 ▶); Sun & Yang (2007 ▶).
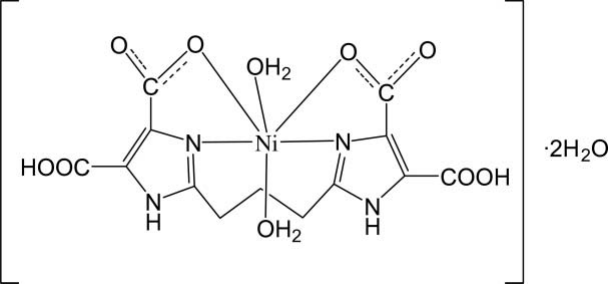


## Experimental

### 

#### Crystal data

[Ni(C_13_H_10_N_4_O_8_)(H_2_O)_2_]·2H_2_O
*M* *_r_* = 481.02
Triclinic, 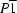

*a* = 8.9852 (18) Å
*b* = 9.4392 (19) Å
*c* = 12.538 (3) Å
α = 108.81 (3)°
β = 92.34 (3)°
γ = 116.18 (3)°
*V* = 882.1 (5) Å^3^
*Z* = 2
Mo *K*α radiation
μ = 1.18 mm^−1^
*T* = 293 K
0.21 × 0.18 × 0.15 mm

#### Data collection

Rigaku Saturn diffractometer
Absorption correction: multi-scan (*CrystalClear*; Rigaku/MSC, 2006 ▶) *T*_min_ = 0.790, *T*_max_ = 0.843
9532 measured reflections
3451 independent reflections
3037 reflections with *I* > 2σ(*I*)
*R*_int_ = 0.025

#### Refinement

*R*[*F* ^2^ > 2σ(*F* ^2^)] = 0.040
*wR*(*F* ^2^) = 0.098
*S* = 1.02
3451 reflections
275 parameters
H-atom parameters constrained
Δρ_max_ = 0.79 e Å^−3^
Δρ_min_ = −0.49 e Å^−3^

### 

Data collection: *CrystalClear* (Rigaku/MSC, 2006 ▶); cell refinement: *CrystalClear*; data reduction: *CrystalClear*; program(s) used to solve structure: *SHELXS97* (Sheldrick, 2008 ▶); program(s) used to refine structure: *SHELXL97* (Sheldrick, 2008 ▶); molecular graphics: *XP* in *SHELXTL* (Sheldrick, 2008 ▶); software used to prepare material for publication: *SHELXTL*.

## Supplementary Material

Crystal structure: contains datablock(s) global, I. DOI: 10.1107/S1600536811024391/wm2496sup1.cif

Structure factors: contains datablock(s) I. DOI: 10.1107/S1600536811024391/wm2496Isup2.hkl

Additional supplementary materials:  crystallographic information; 3D view; checkCIF report

## Figures and Tables

**Table 1:** Selected bond lengths (Å)

Ni1—O5	2.0514 (18)
Ni1—N1	2.060 (2)
Ni1—N3	2.072 (2)
Ni1—O10	2.078 (2)
Ni1—O9	2.093 (2)
Ni1—O1	2.128 (2)

**Table 2:** Hydrogen-bond geometry (Å, °)

*D*—H⋯*A*	*D*—H	H⋯*A*	*D*⋯*A*	*D*—H⋯*A*
O3—H3⋯O2	0.89	1.60	2.485 (3)	176
O7—H7⋯O6	0.89	1.62	2.501 (3)	171
O12—H12*B*⋯O4	0.85	2.57	3.279 (3)	142
N2—H2⋯O12^i^	0.86	1.95	2.802 (3)	171
N4—H4⋯O11^ii^	0.86	1.87	2.721 (3)	170
O10—H10*A*⋯O3^ii^	0.85	2.01	2.853 (3)	171
O9—H9*A*⋯O8^iii^	0.85	1.93	2.781 (3)	176
O9—H9*A*⋯O7^iii^	0.85	2.64	3.157 (3)	121
O11—H11*B*⋯O8^iii^	0.85	2.37	2.884 (3)	120
O9—H9*B*⋯O6^iv^	0.85	1.91	2.762 (3)	175
O10—H10*B*⋯O4^v^	0.85	1.84	2.667 (3)	165
O12—H12*A*⋯O1^v^	0.85	2.26	3.063 (4)	159
O12—H12*B*⋯O10^v^	0.85	2.62	3.198 (4)	126
O11—H11*A*⋯O5^vi^	0.85	1.96	2.762 (3)	157
O11—H11*B*⋯O9^vii^	0.85	2.34	3.100 (3)	148

Symmetry codes: (i) 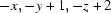; (ii) 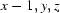; (iii) 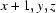; (iv) 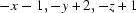; (v) 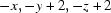; (vi) 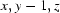; (vii) 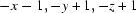.

**Fig. 1.**
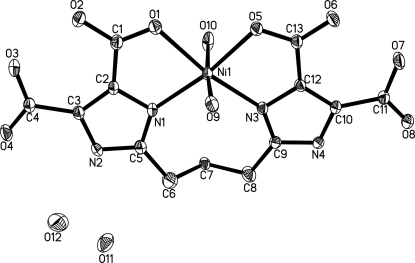
View of the title complex, showing the labelling of the atoms. Displacement ellipsoids are displayed at the 30% probability level. H atoms are omitted for clarity; only one part of the central C atom of the propanediyl unit is shown.

**Fig. 2.**
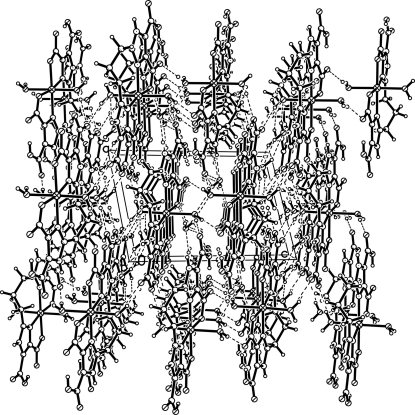
View of the crystal packing of the title comples, showing the three-dimensional structure stabilized by hydrogen bonds (dashed lines).

## supplementary crystallographic information

### Comment

Numerous compounds with metal-organic framework structures constructed from 1*H*-imidazole-4,5-dicarboxylic acid or its derivatives have been synthesized (Baures *et al.*, 2002; Sun & Yang, 2007). To further explore frameworks with new structures, we used 2,2'-(1,3-propanediyl)bis-1*H*-imidazole-4,5-dicarboxylic acid (H_6_pbidc) which has both N-donor and O-donor sites for self-assembly with various metal cations. As a metal source we have used NiCl_2_ and have obtained the title complex [Ni(H_4_pbidc)(H_2_O)_2_]^.^2H_2_O (H_6_pbidc = 2,2'-(1,3-propanediyl)bis-1*H*-imidazole-4,5-dicarboxylic acid), or [Ni(C_13_H_10_N_4_O_8_)(H_2_O)_2_]^.^2H_2_O.

As shown in Figure 1, the Ni^2+^ cation is in a distorted octahedral coordination environment defined by atoms N1, N3, O1, O5 from the tetradentate H_4_pbidc^2-^ anion in equatorial positions and by atoms O9, O10 from water molecules in axial positions. The two imidazole rings are nearly co-planar, with a dihedral angle between the two least-square planes N1, C5, N2, C3, C2 and N3, C12, C10, N4, C9 of 6.8 (2) °. Intramolecular O—H···O hydrogen bonds between the carboxyl/carboxylate groups stabilize the molecular configuration. O—H···O and N—H···O hydrogen bonds between the water molecules and carboxylate O atoms and between imidazole groups and carboxylate O atoms of adjacent molecules consolidate the crystal packing.

### Experimental

A mixture of NiCl_2_ (0.05 mmol), 2,2'-(1,3-propanediyl)bis-1*H*-imidazole-4,5-dicarboxylic (0.05 mmol), methanol (2 ml) and water (2 ml) was placed in a 25 ml Teflon-lined stainless steel vessel and heated at 393 K for 48 h, then cooled to room temperature. Light-green crystals were obtained from the filtrate and dried in air.

### Refinement

The disordered central C atom C7 of the propanediyl unit has been modelled by splitting it into two combined parts (C7 and C7A; ratio 0.531 (6):0.469 (6)). Hydrogen atoms except for those associated with O12 were positioned geometrically and refined as riding atoms, with C–H = 0.97 Å, N–H = 0.86 Å and O–H = 0.85 (H_2_O) and 0.89 (–COOH) Å, and with U_iso_(H) = 1.2 U_eq_(C,N,O). Although water H atoms associated with O12 were located in the difference Fourier map (modelled with an O–H distance constrained to 0.85 Å, and with U_iso_(H) = 1.2 U_eq_(O)), it appears likely, both from the hydrogen bonding scheme and the symmetry-relation of adjacent O12 water molecules *via* inversion centres, that the H atoms of this water molecule are disordered. Nevertheless, the finally obtained model is plausible and we eventually kept these H atoms for refinement.

### Crystal data

**Table:**

[Ni(C_13_H_10_N_4_O_8_)(H_2_O)_2_]·2H_2_O	*Z* = 2
*M**_r_* = 481.02	*F*(000) = 496
Triclinic, *P*1	*D*_x_ = 1.811 Mg m^−^^3^
Hall symbol: -P 1	Mo *K*α radiation, λ = 0.71073 Å
*a* = 8.9852 (18) Å	Cell parameters from 2450 reflections
*b* = 9.4392 (19) Å	θ = 2.6–27.9°
*c* = 12.538 (3) Å	µ = 1.18 mm^−^^1^
α = 108.81 (3)°	*T* = 293 K
β = 92.34 (3)°	Prism, green
γ = 116.18 (3)°	0.21 × 0.18 × 0.15 mm
*V* = 882.1 (5) Å^3^	

### Data collection

**Table:**

Rigaku Saturn diffractometer	3451 independent reflections
Radiation source: fine-focus sealed tube	3037 reflections with *I* > 2σ(*I*)
graphite	*R*_int_ = 0.025
Detector resolution: 28.5714 pixels mm^-1^	θ_max_ = 26.0°, θ_min_ = 2.6°
ω scans	*h* = −11→10
Absorption correction: multi-scan (*CrystalClear*; Rigaku/MSC, 2006)	*k* = −11→11
*T*_min_ = 0.790, *T*_max_ = 0.843	*l* = −15→15
9532 measured reflections	

### Refinement

**Table:**

Refinement on *F*^2^	Primary atom site location: structure-invariant direct methods
Least-squares matrix: full	Secondary atom site location: difference Fourier map
*R*[*F*^2^ > 2σ(*F*^2^)] = 0.040	Hydrogen site location: inferred from neighbouring sites
*wR*(*F*^2^) = 0.098	H-atom parameters constrained
*S* = 1.02	*w* = 1/[σ^2^(*F*_o_^2^) + (0.056*P*)^2^ + 0.3821*P*] where *P* = (*F*_o_^2^ + 2*F*_c_^2^)/3
3451 reflections	(Δ/σ)_max_ < 0.001
275 parameters	Δρ_max_ = 0.79 e Å^−^^3^
0 restraints	Δρ_min_ = −0.49 e Å^−^^3^

### Special details

**Table:**

Geometry. All e.s.d.'s (except the e.s.d. in the dihedral angle between two l.s. planes) are estimated using the full covariance matrix. The cell e.s.d.'s are taken into account individually in the estimation of e.s.d.'s in distances, angles and torsion angles; correlations between e.s.d.'s in cell parameters are only used when they are defined by crystal symmetry. An approximate (isotropic) treatment of cell e.s.d.'s is used for estimating e.s.d.'s involving l.s. planes.
Refinement. Refinement of *F*^2^ against ALL reflections. The weighted *R*-factor *wR* and goodness of fit *S* are based on *F*^2^, conventional *R*-factors *R* are based on *F*, with *F* set to zero for negative *F*^2^. The threshold expression of *F*^2^ > σ(*F*^2^) is used only for calculating *R*-factors(gt) *etc*. and is not relevant to the choice of reflections for refinement. *R*-factors based on *F*^2^ are statistically about twice as large as those based on *F*, and *R*- factors based on ALL data will be even larger.

### Fractional atomic coordinates and isotropic or equivalent isotropic displacement parameters (Å^2^)

**Table:**

	*x*	*y*	*z*	*U*_iso_*/*U*_eq_	Occ. (<1)
Ni1	−0.42402 (4)	0.90838 (4)	0.72525 (3)	0.02527 (13)	
N1	−0.3010 (3)	0.8092 (3)	0.79563 (19)	0.0263 (5)	
N2	−0.1769 (3)	0.6933 (3)	0.8643 (2)	0.0316 (5)	
H2	−0.1639	0.6220	0.8872	0.038*	
N3	−0.6835 (3)	0.7484 (3)	0.69036 (17)	0.0230 (5)	
N4	−0.9581 (3)	0.5817 (3)	0.65436 (18)	0.0251 (5)	
H4	−1.0542	0.4986	0.6509	0.030*	
O1	−0.1678 (2)	1.1022 (3)	0.75887 (18)	0.0361 (5)	
O2	0.0976 (2)	1.1730 (3)	0.8274 (2)	0.0443 (5)	
O3	0.2406 (2)	1.0420 (3)	0.90408 (18)	0.0382 (5)	
H3	0.1934	1.0939	0.8789	0.046*	
O4	0.1688 (3)	0.8133 (3)	0.94778 (18)	0.0394 (5)	
O5	−0.5025 (2)	1.0349 (2)	0.65068 (17)	0.0319 (4)	
O6	−0.7326 (2)	1.0223 (3)	0.56576 (19)	0.0371 (5)	
O7	−1.0474 (2)	0.8392 (3)	0.5275 (2)	0.0451 (6)	
H7	−0.9373	0.9087	0.5364	0.054*	
O8	−1.2265 (2)	0.6084 (2)	0.55412 (17)	0.0356 (5)	
O9	−0.4159 (2)	0.7760 (2)	0.55768 (16)	0.0303 (4)	
H9A	−0.3629	0.7202	0.5567	0.036*	
H9B	−0.3671	0.8430	0.5235	0.036*	
O10	−0.4382 (2)	1.0567 (3)	0.88393 (17)	0.0436 (5)	
H10A	−0.5282	1.0618	0.8968	0.052*	
H10B	−0.3653	1.0951	0.9453	0.052*	
O11	−0.2803 (3)	0.3396 (3)	0.6349 (2)	0.0513 (6)	
H11A	−0.3282	0.2375	0.6319	0.062*	
H11B	−0.3368	0.3486	0.5839	0.062*	
O12	0.1580 (4)	0.5672 (3)	1.0849 (3)	0.0723 (8)	
H12A	0.1773	0.6568	1.1416	0.087*	
H12B	0.2033	0.6225	1.0429	0.087*	
C1	−0.0641 (3)	1.0721 (4)	0.8012 (2)	0.0301 (6)	
C2	−0.1280 (3)	0.9137 (3)	0.8225 (2)	0.0247 (5)	
C3	−0.0494 (3)	0.8432 (3)	0.8655 (2)	0.0260 (6)	
C4	0.1325 (3)	0.9006 (3)	0.9095 (2)	0.0279 (6)	
C5	−0.3264 (3)	0.6761 (4)	0.8214 (3)	0.0331 (6)	
C6	−0.4936 (4)	0.5266 (5)	0.8043 (4)	0.0640 (10)	
H6BC	−0.4708	0.4323	0.7944	0.077*	0.531 (6)
H6BD	−0.5280	0.5511	0.8775	0.077*	0.531 (6)
H6AA	−0.4784	0.4641	0.8480	0.077*	0.469 (6)
H6AB	−0.5309	0.4519	0.7234	0.077*	0.469 (6)
C7	−0.6258 (7)	0.5718 (7)	0.8422 (5)	0.0250 (14)	0.469 (6)
H7A	−0.5750	0.6952	0.8718	0.030*	0.469 (6)
H7B	−0.6568	0.5367	0.9063	0.030*	0.469 (6)
C8	−0.7859 (4)	0.5000 (4)	0.7560 (3)	0.0435 (8)	
H8BC	−0.8907	0.3927	0.7314	0.052*	0.531 (6)
H8BD	−0.7651	0.5553	0.8391	0.052*	0.531 (6)
H8AA	−0.8817	0.4600	0.7918	0.052*	0.469 (6)
H8AB	−0.7941	0.4006	0.6950	0.052*	0.469 (6)
C9	−0.8044 (3)	0.6107 (3)	0.7005 (2)	0.0241 (5)	
C10	−0.9358 (3)	0.7068 (3)	0.6140 (2)	0.0242 (5)	
C11	−1.0812 (3)	0.7141 (3)	0.5620 (2)	0.0284 (6)	
C12	−0.7641 (3)	0.8093 (3)	0.6366 (2)	0.0234 (5)	
C13	−0.6606 (3)	0.9664 (3)	0.6158 (2)	0.0274 (6)	
C7A	−0.6330 (8)	0.4635 (9)	0.7229 (8)	0.0640 (10)	0.531 (6)
H7AA	−0.6791	0.3406	0.6893	0.077*	0.531 (6)
H7AB	−0.5961	0.5056	0.6625	0.077*	0.531 (6)

### Atomic displacement parameters (Å^2^)

**Table:**

	*U*^11^	*U*^22^	*U*^33^	*U*^12^	*U*^13^	*U*^23^
Ni1	0.01820 (19)	0.0280 (2)	0.0328 (2)	0.01094 (15)	0.00339 (14)	0.01571 (15)
N1	0.0189 (11)	0.0294 (12)	0.0302 (12)	0.0123 (10)	0.0013 (9)	0.0103 (9)
N2	0.0277 (12)	0.0314 (12)	0.0435 (14)	0.0188 (11)	0.0040 (10)	0.0174 (11)
N3	0.0191 (11)	0.0269 (11)	0.0259 (11)	0.0117 (9)	0.0059 (9)	0.0126 (9)
N4	0.0183 (11)	0.0232 (11)	0.0323 (12)	0.0076 (9)	0.0048 (9)	0.0121 (9)
O1	0.0262 (10)	0.0364 (11)	0.0517 (13)	0.0140 (9)	0.0055 (9)	0.0255 (10)
O2	0.0227 (11)	0.0410 (12)	0.0694 (15)	0.0095 (10)	0.0035 (10)	0.0302 (11)
O3	0.0232 (10)	0.0449 (12)	0.0467 (12)	0.0179 (10)	0.0009 (9)	0.0159 (10)
O4	0.0331 (11)	0.0454 (12)	0.0407 (12)	0.0250 (10)	−0.0046 (9)	0.0101 (10)
O5	0.0209 (10)	0.0324 (10)	0.0471 (12)	0.0105 (8)	0.0052 (8)	0.0241 (9)
O6	0.0275 (10)	0.0453 (12)	0.0551 (13)	0.0196 (10)	0.0099 (9)	0.0358 (10)
O7	0.0218 (10)	0.0520 (13)	0.0728 (16)	0.0158 (10)	0.0044 (10)	0.0407 (12)
O8	0.0208 (10)	0.0348 (11)	0.0493 (13)	0.0116 (9)	0.0028 (9)	0.0168 (9)
O9	0.0265 (10)	0.0386 (11)	0.0373 (11)	0.0191 (9)	0.0108 (8)	0.0226 (9)
O10	0.0279 (11)	0.0621 (14)	0.0340 (11)	0.0262 (11)	0.0004 (9)	0.0040 (10)
O11	0.0313 (12)	0.0441 (13)	0.0710 (16)	0.0043 (10)	0.0005 (11)	0.0341 (12)
O12	0.090 (2)	0.0575 (16)	0.081 (2)	0.0379 (16)	0.0068 (16)	0.0390 (15)
C1	0.0230 (14)	0.0337 (15)	0.0339 (15)	0.0145 (12)	0.0059 (11)	0.0118 (12)
C2	0.0190 (13)	0.0310 (14)	0.0239 (13)	0.0137 (11)	0.0032 (10)	0.0079 (11)
C3	0.0234 (14)	0.0290 (14)	0.0260 (13)	0.0149 (12)	0.0040 (10)	0.0075 (11)
C4	0.0246 (14)	0.0332 (15)	0.0230 (13)	0.0167 (13)	0.0023 (10)	0.0034 (11)
C5	0.0249 (14)	0.0325 (15)	0.0454 (17)	0.0158 (13)	0.0070 (12)	0.0159 (13)
C6	0.0307 (16)	0.0442 (18)	0.118 (3)	0.0155 (15)	0.0163 (18)	0.036 (2)
C7	0.023 (3)	0.029 (3)	0.030 (3)	0.013 (2)	0.006 (2)	0.019 (2)
C8	0.0361 (18)	0.0466 (19)	0.061 (2)	0.0205 (15)	0.0116 (15)	0.0347 (17)
C9	0.0217 (13)	0.0252 (13)	0.0263 (13)	0.0117 (11)	0.0074 (10)	0.0101 (11)
C10	0.0195 (13)	0.0275 (13)	0.0244 (12)	0.0112 (11)	0.0037 (10)	0.0087 (10)
C11	0.0232 (14)	0.0309 (14)	0.0310 (14)	0.0141 (12)	0.0028 (11)	0.0103 (11)
C12	0.0218 (13)	0.0256 (13)	0.0273 (13)	0.0145 (11)	0.0062 (10)	0.0108 (10)
C13	0.0206 (13)	0.0312 (14)	0.0328 (14)	0.0131 (12)	0.0049 (11)	0.0142 (12)
C7A	0.0307 (16)	0.0442 (18)	0.118 (3)	0.0155 (15)	0.0163 (18)	0.036 (2)

### Geometric parameters (Å, °)

**Table:**

Ni1—O5	2.0514 (18)	O11—H11A	0.8504
Ni1—N1	2.060 (2)	O11—H11B	0.8499
Ni1—N3	2.072 (2)	O12—H12A	0.8500
Ni1—O10	2.078 (2)	O12—H12B	0.8499
Ni1—O9	2.093 (2)	C1—C2	1.466 (4)
Ni1—O1	2.128 (2)	C2—C3	1.358 (4)
N1—C5	1.323 (4)	C3—C4	1.491 (4)
N1—C2	1.376 (3)	C5—C6	1.484 (4)
N2—C5	1.346 (3)	C6—C7A	1.346 (8)
N2—C3	1.368 (4)	C6—C7	1.477 (6)
N2—H2	0.8600	C6—H6BC	0.9700
N3—C9	1.325 (3)	C6—H6BD	0.9700
N3—C12	1.374 (3)	C6—H6AA	0.9702
N4—C9	1.348 (3)	C6—H6AB	0.9698
N4—C10	1.369 (3)	C7—C8	1.496 (6)
N4—H4	0.8600	C7—H7A	0.9700
O1—C1	1.234 (3)	C7—H7B	0.9700
O2—C1	1.290 (3)	C8—C9	1.489 (4)
O3—C4	1.284 (4)	C8—C7A	1.596 (7)
O3—H3	0.8899	C8—H8BC	0.9700
O4—C4	1.222 (3)	C8—H8BD	0.9700
O5—C13	1.258 (3)	C8—H8AA	0.9699
O6—C13	1.254 (3)	C8—H8AB	0.9702
O7—C11	1.303 (3)	C10—C12	1.366 (4)
O7—H7	0.8901	C10—C11	1.475 (3)
O8—C11	1.219 (3)	C12—C13	1.478 (4)
O9—H9A	0.8499	C7A—H6AB	0.9711
O9—H9B	0.8499	C7A—H8AB	1.2792
O10—H10A	0.8497	C7A—H7AA	0.9700
O10—H10B	0.8499	C7A—H7AB	0.9700
			
O5—Ni1—N1	169.24 (8)	C5—C6—H6BD	105.7
O5—Ni1—N3	81.61 (8)	H6BC—C6—H6BD	106.1
N1—Ni1—N3	108.79 (9)	C7A—C6—H6AA	123.7
O5—Ni1—O10	88.48 (9)	C7—C6—H6AA	108.3
N1—Ni1—O10	94.21 (9)	C5—C6—H6AA	108.1
N3—Ni1—O10	89.72 (9)	C7—C6—H6AB	109.8
O5—Ni1—O9	85.09 (8)	C5—C6—H6AB	108.8
N1—Ni1—O9	92.00 (8)	H6BD—C6—H6AB	144.7
N3—Ni1—O9	89.98 (9)	H6AA—C6—H6AB	107.7
O10—Ni1—O9	173.54 (8)	C6—C7—C8	118.4 (4)
O5—Ni1—O1	89.84 (8)	C6—C7—H7A	107.7
N1—Ni1—O1	79.91 (8)	C8—C7—H7A	107.7
N3—Ni1—O1	170.90 (8)	C6—C7—H7B	107.7
O10—Ni1—O1	86.91 (9)	C8—C7—H7B	107.7
O9—Ni1—O1	92.42 (9)	H7A—C7—H7B	107.1
C5—N1—C2	106.1 (2)	C9—C8—C7	118.5 (3)
C5—N1—Ni1	143.02 (19)	C9—C8—C7A	111.2 (4)
C2—N1—Ni1	110.77 (17)	C7—C8—C7A	57.7 (4)
C5—N2—C3	108.5 (2)	C9—C8—H8BC	109.4
C5—N2—H2	125.8	C7—C8—H8BC	131.9
C3—N2—H2	125.8	C7A—C8—H8BC	109.4
C9—N3—C12	106.5 (2)	C9—C8—H8BD	109.4
C9—N3—Ni1	145.05 (18)	C7A—C8—H8BD	109.4
C12—N3—Ni1	108.42 (16)	H8BC—C8—H8BD	108.0
C9—N4—C10	108.6 (2)	C9—C8—H8AA	108.2
C9—N4—H4	125.7	C7—C8—H8AA	108.8
C10—N4—H4	125.7	C7A—C8—H8AA	139.8
C1—O1—Ni1	113.46 (18)	C9—C8—H8AB	107.3
C4—O3—H3	113.8	C7—C8—H8AB	106.4
C13—O5—Ni1	115.03 (17)	H8BC—C8—H8AB	60.8
C11—O7—H7	114.9	H8BD—C8—H8AB	143.2
Ni1—O9—H9A	112.4	H8AA—C8—H8AB	107.1
Ni1—O9—H9B	111.7	N3—C9—N4	110.0 (2)
H9A—O9—H9B	107.0	N3—C9—C8	128.3 (2)
Ni1—O10—H10A	122.8	N4—C9—C8	121.7 (2)
Ni1—O10—H10B	122.2	C12—C10—N4	105.5 (2)
H10A—O10—H10B	113.1	C12—C10—C11	132.8 (2)
H11A—O11—H11B	110.1	N4—C10—C11	121.7 (2)
H12A—O12—H12B	93.0	O8—C11—O7	121.9 (2)
O1—C1—O2	123.1 (3)	O8—C11—C10	120.8 (2)
O1—C1—C2	118.3 (2)	O7—C11—C10	117.3 (2)
O2—C1—C2	118.6 (2)	C10—C12—N3	109.5 (2)
C3—C2—N1	109.7 (2)	C10—C12—C13	131.6 (2)
C3—C2—C1	132.8 (2)	N3—C12—C13	118.9 (2)
N1—C2—C1	117.5 (2)	O6—C13—O5	124.5 (2)
C2—C3—N2	105.5 (2)	O6—C13—C12	119.5 (2)
C2—C3—C4	132.7 (3)	O5—C13—C12	116.0 (2)
N2—C3—C4	121.7 (2)	C6—C7A—C8	120.2 (6)
O4—C4—O3	124.9 (3)	C8—C7A—H6AB	165.8
O4—C4—C3	119.3 (3)	C6—C7A—H8AB	149.0
O3—C4—C3	115.8 (2)	H6AB—C7A—H8AB	151.6
N1—C5—N2	110.2 (2)	C6—C7A—H7AA	107.3
N1—C5—C6	125.8 (3)	C8—C7A—H7AA	107.3
N2—C5—C6	124.0 (3)	H6AB—C7A—H7AA	77.9
C7A—C6—C7	63.7 (4)	H8AB—C7A—H7AA	73.9
C7A—C6—C5	126.5 (5)	C6—C7A—H7AB	107.3
C7—C6—C5	113.9 (3)	C8—C7A—H7AB	107.3
C7A—C6—H6BC	105.7	H6AB—C7A—H7AB	83.1
C7—C6—H6BC	136.7	H8AB—C7A—H7AB	101.6
C5—C6—H6BC	105.7	H7AA—C7A—H7AB	106.9
C7A—C6—H6BD	105.7		
			
N3—Ni1—N1—C5	5.0 (3)	Ni1—N1—C5—N2	177.0 (2)
O10—Ni1—N1—C5	96.1 (3)	C2—N1—C5—C6	−178.3 (3)
O9—Ni1—N1—C5	−85.7 (3)	Ni1—N1—C5—C6	−1.9 (6)
O1—Ni1—N1—C5	−177.8 (3)	C3—N2—C5—N1	−0.5 (3)
O5—Ni1—N1—C2	16.6 (5)	C3—N2—C5—C6	178.4 (3)
N3—Ni1—N1—C2	−178.70 (16)	N1—C5—C6—C7A	29.0 (7)
O10—Ni1—N1—C2	−87.56 (18)	N2—C5—C6—C7A	−149.8 (5)
O9—Ni1—N1—C2	90.65 (18)	N1—C5—C6—C7	−45.1 (6)
O1—Ni1—N1—C2	−1.46 (16)	N2—C5—C6—C7	136.1 (4)
O5—Ni1—N3—C9	−177.6 (3)	C7A—C6—C7—C8	2.0 (5)
N1—Ni1—N3—C9	5.2 (3)	C5—C6—C7—C8	122.5 (4)
O10—Ni1—N3—C9	−89.1 (3)	C6—C7—C8—C9	−100.1 (5)
O9—Ni1—N3—C9	97.3 (3)	C6—C7—C8—C7A	−1.8 (4)
O5—Ni1—N3—C12	1.02 (16)	C12—N3—C9—N4	0.5 (3)
N1—Ni1—N3—C12	−176.13 (15)	Ni1—N3—C9—N4	179.1 (2)
O10—Ni1—N3—C12	89.52 (17)	C12—N3—C9—C8	−177.8 (3)
O9—Ni1—N3—C12	−84.02 (16)	Ni1—N3—C9—C8	0.9 (5)
O5—Ni1—O1—C1	−175.7 (2)	C10—N4—C9—N3	−0.7 (3)
N1—Ni1—O1—C1	1.0 (2)	C10—N4—C9—C8	177.7 (2)
O10—Ni1—O1—C1	95.8 (2)	C7—C8—C9—N3	21.0 (5)
O9—Ni1—O1—C1	−90.7 (2)	C7A—C8—C9—N3	−42.8 (5)
N1—Ni1—O5—C13	163.9 (4)	C7—C8—C9—N4	−157.1 (3)
N3—Ni1—O5—C13	−1.47 (19)	C7A—C8—C9—N4	139.1 (4)
O10—Ni1—O5—C13	−91.4 (2)	C9—N4—C10—C12	0.6 (3)
O9—Ni1—O5—C13	89.2 (2)	C9—N4—C10—C11	−177.8 (2)
O1—Ni1—O5—C13	−178.32 (19)	C12—C10—C11—O8	−177.5 (3)
Ni1—O1—C1—O2	179.6 (2)	N4—C10—C11—O8	0.3 (4)
Ni1—O1—C1—C2	−0.2 (3)	C12—C10—C11—O7	1.2 (4)
C5—N1—C2—C3	−0.4 (3)	N4—C10—C11—O7	179.0 (2)
Ni1—N1—C2—C3	−178.14 (17)	N4—C10—C12—N3	−0.3 (3)
C5—N1—C2—C1	179.5 (2)	C11—C10—C12—N3	177.8 (3)
Ni1—N1—C2—C1	1.8 (3)	N4—C10—C12—C13	−178.8 (3)
O1—C1—C2—C3	178.9 (3)	C11—C10—C12—C13	−0.7 (5)
O2—C1—C2—C3	−1.0 (5)	C9—N3—C12—C10	−0.1 (3)
O1—C1—C2—N1	−1.1 (4)	Ni1—N3—C12—C10	−179.28 (17)
O2—C1—C2—N1	179.0 (2)	C9—N3—C12—C13	178.6 (2)
N1—C2—C3—N2	0.1 (3)	Ni1—N3—C12—C13	−0.6 (3)
C1—C2—C3—N2	−179.9 (3)	Ni1—O5—C13—O6	−178.4 (2)
N1—C2—C3—C4	−178.8 (3)	Ni1—O5—C13—C12	1.5 (3)
C1—C2—C3—C4	1.2 (5)	C10—C12—C13—O6	−2.3 (4)
C5—N2—C3—C2	0.2 (3)	N3—C12—C13—O6	179.3 (2)
C5—N2—C3—C4	179.3 (2)	C10—C12—C13—O5	177.7 (3)
C2—C3—C4—O4	177.4 (3)	N3—C12—C13—O5	−0.6 (4)
N2—C3—C4—O4	−1.4 (4)	C7—C6—C7A—C8	−1.9 (5)
C2—C3—C4—O3	−2.4 (4)	C5—C6—C7A—C8	−103.3 (6)
N2—C3—C4—O3	178.7 (2)	C9—C8—C7A—C6	113.1 (6)
C2—N1—C5—N2	0.6 (3)	C7—C8—C7A—C6	2.0 (5)

### Hydrogen-bond geometry (Å, °)

**Table:**

*D*—H···*A*	*D*—H	H···*A*	*D*···*A*	*D*—H···*A*
O3—H3···O2	0.89	1.60	2.485 (3)	176
O7—H7···O6	0.89	1.62	2.501 (3)	171
O12—H12B···O4	0.85	2.57	3.279 (3)	142
N2—H2···O12^i^	0.86	1.95	2.802 (3)	171
N4—H4···O11^ii^	0.86	1.87	2.721 (3)	170
O10—H10A···O3^ii^	0.85	2.01	2.853 (3)	171
O9—H9A···O8^iii^	0.85	1.93	2.781 (3)	176
O9—H9A···O7^iii^	0.85	2.64	3.157 (3)	121
O11—H11B···O8^iii^	0.85	2.37	2.884 (3)	120
O9—H9B···O6^iv^	0.85	1.91	2.762 (3)	175
O10—H10B···O4^v^	0.85	1.84	2.667 (3)	165
O12—H12A···O1^v^	0.85	2.26	3.063 (4)	159
O12—H12B···O10^v^	0.85	2.62	3.198 (4)	126
O11—H11A···O5^vi^	0.85	1.96	2.762 (3)	157
O11—H11B···O9^vii^	0.85	2.34	3.100 (3)	148

Symmetry codes: (i) −*x*, −*y*+1, −*z*+2; (ii) *x*−1, *y*, *z*; (iii) *x*+1, *y*, *z*; (iv) −*x*−1, −*y*+2, −*z*+1; (v) −*x*, −*y*+2, −*z*+2; (vi) *x*, *y*−1, *z*; (vii) −*x*−1, −*y*+1, −*z*+1.
